# Axillary Artery Approach for Ductal Stenting in Neonates and Infants

**DOI:** 10.1007/s00246-025-03927-0

**Published:** 2025-06-23

**Authors:** Merve Maze Aydemir, Kahraman Yakut, Erman Cilsal, Muhammet Hamza Halil Toprak, Sezen Gulumser Sisko, Erkut Ozturk, Alper Guzeltas, Ibrahim Cansaran Tanidir

**Affiliations:** 1 Department of Pediatric Cardiology, University of Health Sciences, Istanbul Mehmet Akif Ersoy Thoracic and Cardiovascular Surgery Training and Research Hospital, 34303 Istanbul, Turkey; 2 Department of Pediatric Cardiology, University of Health Sciences, Basaksehir Cam ve Sakura City Hospital, Istanbul, Turkey

**Keywords:** Axillary artery, Cardiac catheterization, Ductal stent, Pediatric

## Abstract

Ductal stenting in patients with unusual arterial duct anatomy presents a challenge. The axillary artery (AA) approach provides a safe and effective alternative for access, particularly when conventional femoral routes fail. A retrospective analysis was conducted on patients with ductal-dependent pulmonary blood flow who underwent ductus arteriosus (DA) stenting via the AA route between November 2018 and December 2022 at two tertiary cardiac centers. Demographic and procedural data were reviewed. The study included 39 patients, mostly neonates and infants with complex DA anatomy. The median age was 11 days (IQR7–15 days), and the median weight was 3000 g (IQR 2700–3400 g). Pulmonary atresia was diagnosed in 30 patients, and 9 had antegrade flow. The AA approach was the primary choice in 15 patients (38%) and used as a secondary option after femoral failure in others. Ultrasound-guided puncture was performed in 15 cases. Thirty-five out of 39 patients (89%) had procedural success, which is defined as stent insertion without the requirement for prostaglandin infusion. The median fluoroscopy and procedure times were 17.1 and 68 min, respectively. Including one temporary brachial plexus injury and access-related events, the overall complication rate was 22% (8/35). Most complications occurred early in the learning curve and were managed without long-term sequelae. The AA is a safe and effective alternative for stenting complex or tortuous ductus arteriosus, especially when femoral access is unsuccessful. It provides favorable alignment and procedural control without significantly increasing procedure or fluoroscopy times.

## Introduction

Stent implantation of ductus arteriosus (DA) has been reported as an alternative to Blalock–Taussig (BT) shunt for palliation in patients with ductal-dependent pulmonary blood flow (PBF) [1, 2]. Recent studies comparing both procedures showed that DA stenting is preferable over using a BT shunt in terms of survival to next-stage surgery, early postprocedural hemodynamic stability, and shorter intensive care unit and hospital stays [3, 4].

The DA can be variable in its systemic arterial origin, diameter, tortuosity, and insertion in patients with ductal-dependent PBF. These can cause particular challenges, and access via the femoral artery or vein may not be possible or challenging with a vertical origin DA. After the 4–5 French sheath is introduced into the femoral artery in newborns and infants, complications such as diminished distal pulses, ischemia, and thrombosis in the lower extremities may occur due to their smaller vessel size compared to the carotid artery (CA) or AAs [2]. The CA and AA accesses have been popular alternatives for stenting in some infants with challenging DA anatomy. AA approach allows quick access to the duct and direct alignment with the DA, like carotid access [5, 6]. The current study aims to describe our experience with DA stenting using the AA approach.

## Methods

A retrospective analysis was performed on infants and neonates who underwent AA catheterization between November 2018 and December 2022 from two tertiary cardiac centers. Patients with ductal-dependent PBF and a stent implanted in the DA were included in the study. The patients’ medical records were reviewed to verify demographic and procedural details.

### Pre-procedure

The diagnosis is made after echocardiographic evaluation. A council decides to place DA stents in all patients with the participation of cardiovascular surgeons. Prostaglandin infusions were stopped 24–48 h before the procedure. DA blood flow was checked frequently with saturation and echocardiographic evaluations. When the saturation decreased under critical levels (< 70%), the prostaglandin infusion was started again. Also, the time interval between the ceasing of the prostaglandin infusion and ductal constriction was recorded. So that, we note how soon the DA constricts. We stopped prostaglandin again, according to the time of the ductus constriction determined previously, before the procedure. All procedures were performed under general anesthesia while the patients were intubated.

### Procedure

Initial arterial access was performed in either the femoral route (artery or vein) or AA (for some patients); the femoral approach can be the first access; if additional interventional procedures are needed, or after the aortogram from the femoral approach, we could decide on AA stenting because of the complex DA anatomy. The choice of vascular access was determined based on arch-sidedness, the anatomical relationship of PDA insertion to head-neck arteries, ventriculo-arterial relationship, anatomical localization of the DA and existing a ventricular septal defect (VSD), which may allow advancing catheters safely from femoral vein to aorta.

For the AA approach, the choice of vascular siding (left or right side) was determined based on arch-sidedness, and the anatomical relationship of DA insertion to head-neck arteries. After an aortogram or if the patient had a cardiac computed tomography evaluation, interventionalists are sure of DA localization and its relationship with the AA. Always remember to check the existence of an aberrant subclavian artery. However, if the initial attempt was unsuccessful, the contralateral axillary artery was used as an alternative. During this transition, the patient’s position remained unchanged, and the operator shifted to the opposite side to gain vascular access. Once access was successfully obtained, the procedure was continued from the patient’s right side for consistency and optimal maneuverability.

The head is slightly turned to the right/or left, and the patient’s arm was abducted for the angle of the left upper limb to the thorax has to be about 120–140°; an extreme extension of the left arm leads to expose the axillary area. Towels were placed under the arm for support. A 4Fr short sheath was inserted into the AA with one of three of these methods: first direct percutaneous access, second using ultrasound guidance, and third placing a wire target from the femoral sheath [1].

If a femoral sheath was placed, the initial angiogram was performed to define DA anatomy and its relation to the AA. In some cases, the procedure was performed first with AA puncture due to echocardiographic or CT evaluation. Intravenous heparin (100 U/kg) with ACT was maintained at > 200 s, and antibiotic prophylaxis was performed with cefazolin for 24 h. Then a 0.014″ ChoiCE Extra Support Guidewire with hydrophilic coating (Boston Scientific Corporation, MN) or Hi-Torque Pilot 50 guidewire (Abbott Vascular, Santa Clara, CA, USA) was advanced into a branch pulmonary artery or the right ventricle if antegrade flow was present until a suitable position was established. In some cases, the buddy-wire technique was used to assist in straightening the DA, particularly when the anatomy was highly tortuous. A floppy or hydrophilic wire was typically used initially to navigate the DA and reduce tortuosity. Once the DA was partially straightened, a coronary stiff wire was advanced to provide additional support. In suitable anatomies, a stiff wire could be used as the first choice. The removal of the buddy wire depended on the sheath size: in cases where a 5F sheath was used, the buddy wire could remain during stent advancement and be removed afterward; however, with a 4F sheath, the buddy wire had to be removed before stent advancement due to the limited inner diameter.

**Fig. 1:**
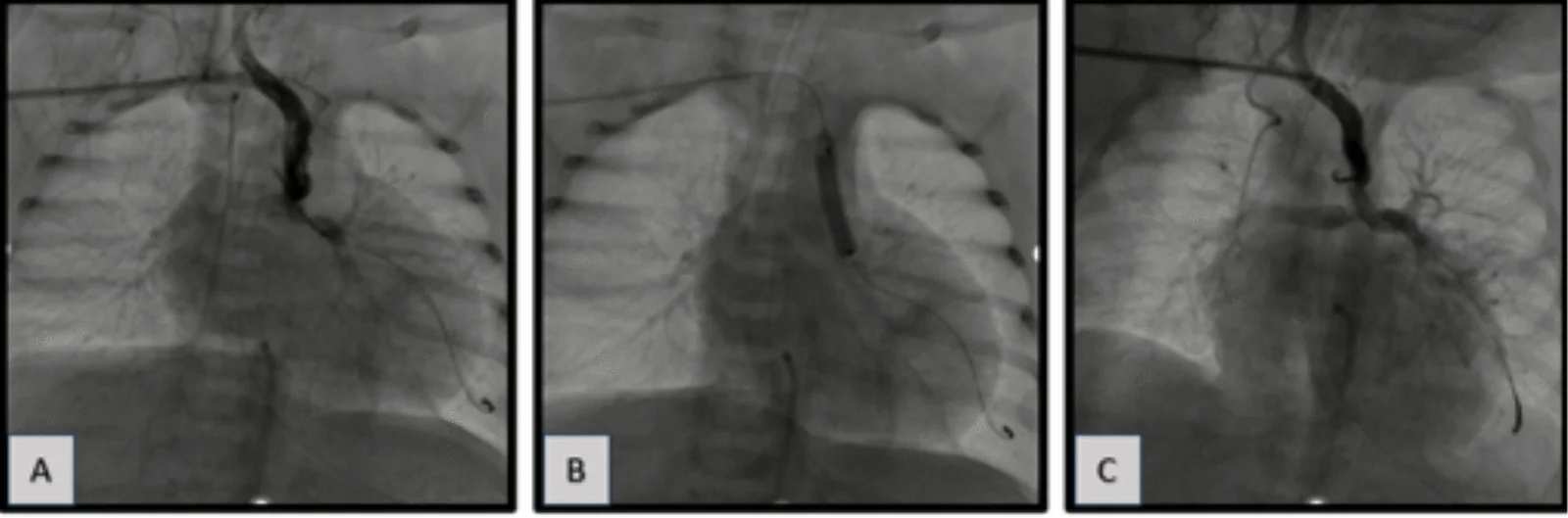
AP projections demonstrating in **A** 0.014 extra support guidewire with hydrophilic coating wire was inserted through the DA into the left pulmonary artery. In **B** positioning a coronary stent across the entire DA length over a coronary guidewire. In **C** illustrating angiographic findings and stent placement post‐intervention

Following straightening of the DA, a 2 mm diameter, 15–20 mm length coronary balloon was advanced over the wire to reassess and accurately measure the true length of the DA. This was necessary because the functional length often changed after straightening. While the balloon was positioned within the DA, contrast injection was performed to delineate the landing zones and guide stent sizing. Although not routine, balloon inflation was occasionally used to assess or restore ductal patency in cases where flow was diminished during wire manipulation. Then, a coronary bare stent (Ephesos II stent system, Alvimedica, Turkey) was delivered over the wire and implanted into the DA. The patient’s body weight and the existence of antegrade pulmonary blood flow were taken into consideration when choosing the stent diameter. A 4.5 mm stent was utilized for patients with pulmonary atresia who weighed more than 4 kg, 4.0 mm for those who weighed 3.5–4 kg, 3.5 mm for those who weighed 2.5–3.5 kg, and 3.0 mm for those who weighed less than 2.5 kg. In cases with additional antegrade pulmonary flow, the chosen stent diameter was typically 0.5 mm less than the norm, but still at least 3.0 mm. Once the optimum stent position was established, the stent was deployed into the DA (Fig. 1). The process was repeated with another stent or stents, until the stent covered both the aortic and pulmonary end of the DA. Exit angiography assessed the stent, branch pulmonary arteries, and aortic arch.

### Post-procedure

After the procedure, all patients were monitored in the pediatric cardiac intensive care unit. Brachial and radial pulses in the ipsilateral arm were monitored for 24 h. Pulses and perfusion in the upper part of the body were assessed shortly after the procedure. After successful stent placement, continuous heparin infusion (20 Units/kg/hour) was continued for at least 24 h. Acetylsalicylic acid (5 mg/kg/day, once daily) and clopidogrel (1 mg/kg/day, once daily) were used after the procedure, during heparin infusion. The team re-evaluated the extremity and pulses the next day, and a transthoracic echocardiogram was performed within 24 h. No other specific neurologic assessment was performed to assess the brachial plexus.

### Statistical Analysis

Data from the two centers were summarized as a single group. Statistical analyses were performed using SPSS for Windows (Version 21, SPSS Inc., USA) software package. Median with interquartile range (IQR) was used to describe continuous data, whereas absolute count with percentage was used for categorical data. Data were analyzed for correlation between the scores and outcome using Spearman’s rho. Chi-square and Mann–Whitney *U* tests were used to compare groups, and the Wilcoxon test was used for repeated consecutive measurements. Statistical significance was set to *p* = 0.05.

## Results

Thirty-nine patients, 21 female (%54) and 18 male (%46), were included in this study. The median age was 11 days (IQR 7–15 days), and the median body weight was 3000 gr (IQR 2700–3400 gr). Nine patients had pulmonary antegrade flow, and 30 patients were diagnosed with pulmonary atresia. Demographic data and the primary diagnoses are summarized in Table 1.

**Table 1:** Demographic data for the cohort

	Median (IQR)
Median age at procedure (days)	11 (7–15)
Median weight (gr)	3000 (2700–3400)
Female: Male	21(54)
Primary diagnosis	
DORV-PA	10
VSD-PA	8
TA-PA	6
DORV-PS	3
IVS-PA	3
Ebstein-PA	2
DILV-PA	2
cc-TGA-PS	2
TA-PS	2
AVSD-TOF	1

*AVSD* atrioventricular septal defect, *ccTGA* congenitally corrected transposition of the great arteries, *DILV* double inlet left ventricle, *DORV* double outlet right ventricle, *IVS* intact ventricular septum, *PA* pulmonary atresia, *PS* pulmonary stenosis, *TA* tricuspid atresia, *TOF* tetralogy of fallot

The expected ultimate physiology was single ventricle for 25 patients (64%) and biventricle for 14 patients (36%). Balloon atrial septostomy was performed in 4 patients at the same time before the stenting procedure.

The AA was the first choice used in 15 patients (38%), and in other patients, it was preferred after the femoral artery/vein failed. In 21/39 patients (54%), the AA was punctured with direct palpation; in 15/39 patients (38%) with the USG guidance, and for 3/39 of them (8%), the wire target approach was preferred. In 36 patients (92%), the procedure was performed from the right AA and 3 patients from the left AA.

The DA originated from under the arch in 38 patients, and one of the patients had two DA, one of the DA from the innominate artery and the other vertical one from under the arch. The tortuosity index was classified as Type I (straight), Type II (one turn), and Type III (many turns) using the Qureshi et al. [7] classification method. PDA type-3 was multiple angled in 31 patients (79%), and 32 patients had left aortic arch. In 6 patients, a jump was observed during the stent release phase.

When balloon atrial septostomy patients were excluded, and only the procedural data of patients who underwent isole AA stenting procedures were evaluated, the median procedure time was 68 min (IQR 55–75 min). Median scope time was 17.1 min (IQR 10–25.2 min).

In the patient group where the first choice was the axillary artery, the mean procedure time was 50 min, and the fluoroscopy time was 15.4 min; in the patient group where the first choice was the femoral artery, the mean procedure time was 70 min and the fluoroscopy time was 18.5 min. The procedure and fluoroscopy times were similar (*p* > 0.05). Median amount of contrast was 7.1 cc/kg (6–7.5 cc/kg).

The procedure’s success was defined by successful stent implantation without the need for prostaglandin. Consequently, the procedure was deemed unsuccessful in 3 patients due to the inability to traverse the ductus caused by DA collapse, and in 1 patient due to embolization occurring before the stent could be positioned and opened. The AA approach was completed in 35 out of 39 patients, resulting in an 89% success rate.

Among 35 patients, a single stent was used in 21, and two were used in 14 patients. The median length of the stents was 16 mm (ranging from 8 to 19 mm). The average oxygen saturation levels were 70 and 90% before and after the procedure. No mortality was observed within the initial 72 h following the procedure. The median length of hospital stay was 14.5 days (IQR 10–20). The median follow-up duration was 29.5 months (IQR 20–40). The median follow-up duration was 29.5 months (ranging from 6 days to 60.2 months). Procedural and device data are summarized in Table 2.

**Table 2:** Procedural and device data of the implanted stents (*n* = 35)

Expected ultimate physiology	
Single ventricle, *n* (%) Biventricular, *n* (%)	22 (62%) 13 (37%)
Duration of intervention, median (IQR) (*n* = 31)	68 min (55–70)
Fluoroscopy time, median (IQR) (*n* = 31)	17.1 min (10–25.2)
AA route as first choice, *n* (%) AA after femoral artery failure, *n* (%)	15 (42%) 20 (57%)
AA with direct palpation, *n* (%) USG guidance, *n* (%) Wire target, *n* (%)	17 (51%) 14 (42%) 2 (6%)
Balloon atrial septostomy, *n* (%)	4 (11%)
Amount of contrast, median (IQR)	7.1 cc/kg (6–7.5)
PDA from under the arch, *n* (%) PDA from innominate artery, *n* (%)	34 (97%) 1 (3%)
PDA type-3 multiple angled, *n* (%)	29 (82%)
Patients with left aortic arch, *n* (%)	28 (80%)
Patients with jump during stent release phase, *n* (%)	6 (17%)
Patients with complications, *n* (%)	8 (22%)
Stent malposition, *n* Pseudoaneurysm, *n* Brachial plexus paralysis, *n*	4 3 1
Single stent, *n* (%) Two stents, *n* (%)	21 (60%) 14 (40%)
Stent length, median (min–max)	16 mm (8–19 mm)
Average saturation	
Before procedure (%) After procedure (%)	70% 90%
Mortality in the first 72 h after procedure, n	0
Discharge time, median (IQR)	14.5 days (10–20)
Follow-up time, median (IQR)	29.5 months (20–40)

Percentages in Table 2 are calculated based on the subset of patients with successful stent implantation (*n* = 35), whereas percentages in the text refer to the total study population (*n* = 39).

Poor limb perfusion, mortality and thromboembolic events were considered as major complications. In patients with successful procedures, access-related complications occurred in 11% (4/35) of cases (three pseudoaneurysms and one brachial plexus paralysis). Additionally, procedural complications such as stent malposition were observed in 11% (4/35) of patients. Overall, the total complication rate was 22% (8/35).

Two of the patients with stent malposition were referred to surgery urgently under cardiopulmonary resuscitation; the stents were removed, and a BT shunt was performed. One of these patients had to be placed on ECMO support on the first day after surgery due to low cardiac output. After ten days of ECMO follow-up, the patient was lost due to sepsis and multi-organ failure. The other patient, on the other hand, was discharged in good health after the BT shunt. The sent malposition was minimal in the other two patients; the problem was like milking. The saturation increased to 90%, and we decided to follow up with the patient. During follow-up, due to desaturation, a central shunt was required in one of these patients one day after stent implantation and in the other 19 days later.

The sheath was inserted under ultrasound (USG) guidance in all three patients who developed pseudoaneurysm. Two patients were treated surgically, and one by interventional radiology. One patient died due to sepsis ten days after the procedure. Others are currently doing well with no issues in follow-up [8].

Brachial plexus paralysis was detected in one patient the day after the procedure. The diagnosis was made clinically based on the absence of arm movement, and stroke was ruled out following evaluation by a pediatric neurologist and a physical medicine specialist. The procedure was performed under general anesthesia without the use of local anesthetics. Given the patient’s positioning and similar rare cases observed in our institution, a positional neuropathy was suspected. The condition improved with physiotherapy, and there were no residual findings at 27 months of follow-up.

## Discussion

Stenting of the DAs as an alternative to surgical shunts has become popular since the initial reports from Gibbs et al. [9]. But the DAs in patients with severe right heart obstructive lesions may have a very challenging anatomy, and these vertical and tortuous DAs have been risk factors for procedural failure from femoral route [5, 10]. If the femoral route fails, options are the axillary approach, carotid approach, and surgical shunt. In countries where newborn shunt surgery has high mortality, alternative transcatheter routes should be kept in mind. Here, we share our experience with the axillary approach for PDA stenting in duct-dependent pulmonary circulation patients.

CA and AA approaches can be chosen as alternative routes for stenting challenging DAs. Vertical DA stenting involves a notably straighter approach angle from the CA compared to the femoral approach. This enhanced alignment simplifies the passage of wires and stents [11]. However, some complications such as thrombus, pseudoaneurysm, and aortic dissection have been reported [5, 6]. Also, there are still some concerns about brain development and cerebral perfusion in the long term. A crucial benefit of the AA approach is that the AA is not an end-artery. Perfusion to the distal AA is primarily maintained through the subscapular artery, which gives rise to the circumflex scapular and thoracodorsal arteries. This network plays a crucial role in collateral circulation and ensures continued perfusion to the upper limb [12]. Another disadvantage of the CA approach is positioning the patient. This can be handled by the flip method, but it can still be challenging for patients who need additional interventions, like balloon atrial septostomy. Hence, in the AA approach from the right AA, interventionalists do not need to change the patient/table position or his/her position. Due to these considerations, our institutions favor the AA approach more frequently.

Studies in the literature present complication-free and 100% successful outcomes [2, 10]. In our study, the procedure was successful in 89%, the stent was implanted by deploying it in the DA, and the patient no longer required PGE. Moreover, while the number of complications decreased with experience, some cases required urgent intervention, including surgical repair and resuscitative measures. Despite these challenges, no patients experienced long-term blood flow issues after the procedure, supporting the axillary artery as a viable option for peripheral perfusion.

The utilization of the AA method for vertical DA stenting has been demonstrated to result in reduced fluoroscopy exposure and shorter overall procedural duration compared to the femoral approach [1]. In our procedural experience, the median procedure and fluoroscopy durations were 68 min and 17.1 min, respectively. These findings align with previous studies, which reported procedural times ranging from 51 to 138 min and fluoroscopy times from 15.5 to 22 min [2, 5, 10]. In our series using the AA as the primary or rescue approach, we found that patients who were first approached via the femoral route and then switched to AA access had lengthier procedural and fluoroscopy delays. Nevertheless, there was no statistically significant change. The viability and usefulness of the AA method are further supported by our recent findings, which indicate that even in cases where femoral access failed, the use of axillary access produced acceptable procedure and fluoroscopy times. Because of its advantageous alignment and access angle, we recommend using the axillary route as the first line of treatment when computed tomography or echocardiography revealed a vertical and tortuous DA that was inappropriate for femoral access.

The axillary approach carries inherent risks, as described in the literature, including dissection, hematoma, and pseudoaneurysm [4, 5]. Breatnach et al. [2] reported a complication rate of 20%, detailing instances such as partial dissection to the subclavian artery, a pseudoaneurysm in the right axillary artery, and ductal spasm that required conversion to an emergency pulmonary valvuloplasty and BT shunt. Bauser-Heaton et al. [6] reported a complication rate of %20 in the CA/AA group together, including arrhythmia, stent malposition, sheath dislodged during the procedure, access site injury, and vascular thrombosis at the non-access site (ductal stent/pulmonary artery thrombosis) and an aortic dissection at the time of stent placement from the CA approach. Lee et al. [5], on the other hand, disclosed a complication rate of 28%, including intra-procedural stent thrombosis and a small left axillary pseudoaneurysm. Polat [10] reported a 10% complication rate in the axillary access group. However, it should be noted that the elevated pulmonary blood flow requiring antifailure treatment was related to procedural factors, such as stent size and flow dynamics, rather than the access route itself. Our total complication rate was 22%. Stent malposition occurred in four cases, with two requiring surgical shunt placement. To avoid this, surgical backup should be prepared during procedures, and close follow-up of oxygen saturation is recommended. Three cases of pseudoaneurysm were observed, two of which required surgical correction, while one was managed with ultrasound-guided percutaneous thrombin injection. Based on our institutional experience, we recommend several preventive strategies to reduce vascular complications, including the use of hydrophilic-coated sheaths, hand-made angulation of the sheath, pre-dilatation before sheath introduction, wetting the sheath. While these techniques are not currently supported by specific guidelines or randomized trials, similar procedural modifications—such as sheath preparation, size selection, and ultrasound-guided access—have been reported in previous studies as part of expert interventional practice [2, 5, 6]. One case of brachial plexus paralysis was successfully treated with physiotherapy, with no residual findings after 27 months of follow-up. Early awareness and timely physiotherapy are crucial in avoiding long-term deficits from this complication (Table 3).

**Table 3:** Complications and precautions (*n* = 35)

Complication	*N*	Outcome	How to avoid
Stent malposition	4	Surgical shunt (*n* = 2)	Surgical backup up ready during procedures Close follow-up of the saturation
Pseudoaneurysm	3	Surgical correction (*n* = 2) USG-guided percutaneous injection of thrombin (*n* = 1)	Using hydrophilic-coated sheaths Hand-made angulation for the sheath Predilatation before introducing the sheath Wetting the sheath Surgical cutdown
Brachial plexus paralysis	1	It was improved with physiotherapy; there is no finding after 27 months of follow-up	Early awareness and physiotherapy

In the Polat report [10], the AA was successfully localized using a wire target approach. According to Breatnach et al. [2], the method may lead to fewer punctures and a lower likelihood of hematoma formation. The drawback is that it necessitates a second femoral artery puncture, endangering that vessel. Therefore, they did not favor this method. Despite this, they reported that one patient developed a small pseudoaneurysm in twenty patients. We strongly discourage unnecessary arterial procedures, considering the age range of the individuals receiving our intervention. Nevertheless, we suggest that in situations of vertically originated and tortuous DA presentations, sometimes it would be advantageous to have a second entry point via the femoral route. We are especially aware of the potential for some difficulties, such as spasms brought on by the guidewire passing via the AA, and as such, we prefer using other access methods for the initial angiography.

Straightening the DA is often a critical step before stent implantation, particularly in cases with a tortuous and vertical course. In our experience, attempting to deliver a stent through a significantly curved duct is technically challenging and carries a higher risk of malposition or prolapse. While some operators advocate preserving the natural ductal configuration using a single soft wire to minimize the risk of stent jumping or kinking, we have found that partial straightening—achieved with stiff wires or, when necessary, the buddy-wire technique—facilitates safer and more accurate stent placement. Most complications in our cohort, such as malposition or jump, were associated with failure to achieve adequate alignment of the duct or inflow route. For vertical and tortuous ducts, we believe the AA route provides a more favorable angle for accessing the DA and minimizing stent-related complications.

In reality, depending on the conditions of each patient, we have chosen different tactics in our practice. This involves switching to the axillary route when the femoral approach fails, using the wire-target technique for others, and gaining direct axillary access for some patients (aided by ultrasound guidance or direct palpation). In our patient cohort, those experiencing complications affecting the vasculature at the intervention site did not suffer long-term effects. Specifically, three survivors with pseudoaneurysm and one with plexus paralysis recovered without enduring lasting consequences. Despite inserting a catheter in a patient with brachial plexus paralysis for the first time under ultrasound guidance, subsequent ultrasound scans conducted post-diagnosis did not reveal any hematoma. This experience underscored the possibility of such complications arising without definitive causal identification.

In the existing literature, DA stent implantation via the AA route has been documented to achieve a 100% success rate, regardless of the utilization of ultrasound guidance (USG) [5, 10, 13]. The operator’s level of expertise appears to be a factor in whether this result is achieved. High levels of hand–eye coordination are required to see the needle’s entry into the vessel in real-time. Through consistent practice, this skill can be mastered. Notably, two centers were engaged in our study; one chose USG, while the other did not. The success rates of the two methods were strikingly comparable.

Despite being one of the considerably substantial series delineating ductal stenting through the AA to date, this study inevitably harbors certain limitations. The retrospective design, relatively small patient cohort, and lack of routine post-procedural ultrasonographic evaluation in all cases limit the generalizability of our findings regarding both procedural success and complication rates. Furthermore, time intervals were reported as total procedure and fluoroscopy durations, measured from initial vascular access to stent deployment. The more precise interval from arterial puncture to wire placement within the PDA was not recorded, which may have provided a better assessment of technical efficiency. This omission is acknowledged as a limitation and will be incorporated into the design of future prospective studies.

## Conclusion

In conclusion, the AA approach seems to be a safe and efficient substitute for PDA stenting, especially in cases where femoral access is unsuccessful or in anatomical situations including the vertical and complicated ductus. The technique’s feasibility was further supported by the acceptable results obtained in rescue situations, even with slightly longer operation and fluoroscopy periods. There is a learning curve, and more extensive, multicenter research is necessary to confirm these results.

## Data Availability

No datasets were generated or analysed during the current study.
